# Oral Route Infection by *Trypanosoma cruzi:* From the Beginning to the Present Day

**DOI:** 10.3390/pathogens15010047

**Published:** 2026-01-01

**Authors:** Sebastián Zambrano, Kurt Montoya, Alejandro Avalos, Bessy Gutiérrez, Juan San Francisco, José Luis Vega, Jorge González

**Affiliations:** 1 Molecular Parasitology Unit, Medical Technology Department, Faculty of Health Sciences, University of Antofagasta, Antofagasta 1270300, Chile; sebastian.zambrano.medina@ua.cl (S.Z.); kurt.montoya.rojas@ua.cl (K.M.); alejandro.avalos@uantof.cl (A.A.); bessy.gutierrez@uantof.cl (B.G.); juan.sanfrancisco@uantof.cl (J.S.F.); 2 Department of Basic Sciences, Faculty of Sciences, Santo Tomás University, Antofagasta 1243161, Chile; 3 Department of Physiology, Faculty of Biological Sciences, University of Concepción, Concepción 4070409, Chile; joseluisvega@udec.cl; 4 Center for Immunology and Biomedical Biotechnology of Antofagasta, University of Antofagasta, Antofagasta 1270300, Chile

**Keywords:** *Trypanosoma cruzi*, oral infections, current knowledge

## Abstract

*Trypanosoma cruzi* is the causative agent of Chagas disease, which affects 6–7 million people worldwide. Although the possibility of oral transmission was first scientifically suggested in 1913, it was not until 1968 that the first confirmed cases of human infection via food consumption were reported. This long gap contributed to the widespread perception that oral transmission was a rare or incidental event. Over the past two decades, significant advances have been made in understanding the biological and clinical aspects of oral transmission, including the molecular mechanisms by which metacyclic trypomastigotes establish infection via the digestive route. Experimental studies in murine models have further deepened our knowledge of the biology and pathogenesis of oral infection. Concurrently, multiple outbreaks of *T. cruzi* infection through contaminated food and beverages have been reported across Latin America, providing valuable insights into the molecular epidemiology and clinical characteristics of this transmission route. Moreover, experimental evidence has shown that the consumption of meat from animals infected during the acute phase can also lead to *T. cruzi* infection, highlighting carnivory as a potential alternative transmission mechanism. This review aims to comprehensively analyze oral infection by *T. cruzi*, considering clinical and epidemiological data, parasite biology, and findings from murine experimental models. Strategies for controlling foodborne transmission of Chagas disease are also discussed.

## 1. Introduction

Chagas disease is a Neglected Tropical Disease first described by Carlos Chagas in 1909 [1]. It is endemic in Latin America, present in 22 countries across the Americas [2,3]. However, because of population migrations, carriers of infection or disease are now found throughout the world, which is why Chagas is considered a global public health problem [4]. Chagas disease is caused by a flagellated protozoan called *Trypanosoma cruzi* (*Kinetoplastida, Trypanosomatidae*) [5]. According to the World Health Organization (WHO), there are 6 to 7 million people infected with *T. cruzi* worldwide [6]. Approximately 75 million people are at risk of acquiring the infection, and more than 10.000 people die each year from the disease [6].

When transmission occurs via the vector route or oral route (ingestion of food contaminated with triatomine droppings), the infective form is the metacyclic trypomastigote (MT). This form invades nucleated cells and differentiates into amastigotes, which divide by binary fission. The amastigotes subsequently differentiate into blood trypomastigotes (BT), which leave the cell to enter the bloodstream, spread to other sites in the organism, and can eventually be ingested by triatomines when they feed on a *T. cruzi*-infected host [7,8].

**Figure 1:**
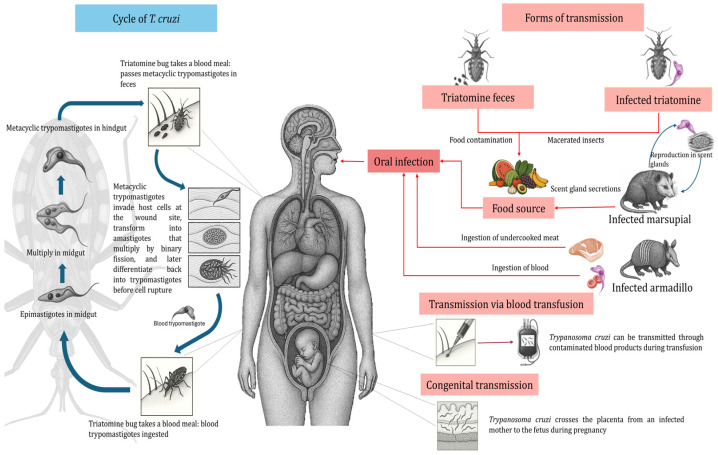
Life cycle of *Trypanosoma cruzi*, including different transmission mechanisms.

When infection occurs by post-transfusion mechanisms, by the transplacental route, transplantation of organs from infected persons, or consumption of meat or blood from infected animals, the infective form is the BT. This stage, like the MT, is able to invade nucleated cells, differentiate within them into the amastigote stage, proliferate intracellularly, and then differentiate into the BT stage. The latter exit the cell, disseminate via the bloodstream, and can also be infective for triatomines when they feed on *T. cruzi*-infected hosts [8] (Figure 1).

In recent decades, however, oral transmission has become an increasingly important mechanism in the epidemiology of Chagas disease [9,10,11]. Notably, 70% of the ~1000 acute Chagas disease cases documented in the first ten years of this century were the result of oral transmission [12,13]. In fact, oral infection with *T. cruzi* is currently the most important transmission route in Brazil. Several outbreaks have been documented in the Amazon region and in some states of the northeast and south of Brazil, and this trend has been on the rise in recent years [14]. Approximately 3.060 cases of acute Chagas disease were reported in Brazil between 2007 and 2019, with a predominance in the north region (94.4%). Pará state had the major percentage of cases (74.54%), most attributed to the ingestion of contaminated food [15]. This trend may be explained by several factors. One important factor is based on human activities: through predation, invasion, and deforestation of jungles and forests, humans have disrupted the ecological balance. This has reduced the diversity of wild mammals that serve as food sources for triatomines. Consequently, triatomines, which are typically wild or peridomestic, have started to invade homes in search of new food sources (blood meals). This increases the chances of food contamination, especially of homemade fruit juices, which have been the most frequent source of infection in the majority of documented oral transmission outbreaks [10]. Therefore, although oral transmission of *T. cruzi* infection is not a global problem, it is a situation of high epidemiological risk in Brazil and countries in northern South America where domestic or peridomestic vectors still exist. On the other hand, the growing export and widespread consumption of açaí pulp in different parts of the world raise the possibility of a more widespread epidemiological risk for this route of infection of the parasite.

The objective of this review is to comprehensively analyze oral infection by *T. cruzi*. Then, we will describe the parasite evolutionary context, proposing oral infection as an ancestral mechanism of infection and analyzing the main clinical and epidemiological findings, as well as parasitic biology and experimental oral infection in the murine model. The present and future challenges in the perspective of strategies for controlling oral infection by *T. cruzi* will also be analyzed.

## 2. The Beginning of Oral Transmission by *T. cruzi*

*T. cruzi* exhibits a high degree of genetic heterogeneity that has been associated with the observed variability in clinical manifestations, geographical distribution, and preferential parasite–vector interactions. The parasite is classified into at least seven discrete typification units (DTUs): TcI–TcVI and TcBat [16]. Some DTUs have been tentatively associated with particular geographic distributions, transmission cycles, or clinical outcomes, but these patterns are not yet fully understood [16,17,18,19,20]. It has been suggested that the infective form of the parasite (e.g., BT vs. MT) and the route of infection (oral vs. other routes) can differentially modulate the course of *T. cruzi* infection in mice [20,21].

*Trypanosoma cruzi* is an ancient parasite. The origin of *T. cruzi*, as well as other kinetoplastid species, has been inferred primarily through molecular phylogenetic analyses based on ribosomal RNA gene sequences. In fact, phylogenetic analysis of 18S rRNA sequences indicates that the *Trypanosoma brucei* clade diverged from the *T. cruzi* clade approximately 100 million years ago, following the separation of Africa, South America, and Euramerica [22].

A Bayesian coalescent analysis has estimated that TcBat could have emerged about 688,000 years ago, whereas TcI emerged around 614,000 years ago. These dates suggest that the current diversity of *T. cruzi* DTUs is relatively recent in evolutionary terms, occurring during the late Pleistocene, and long after the initial radiation of the genus *Trypanosoma* [23].

These temporal estimates support a scenario in which ancient divergence events at the genus level were followed much later by more recent diversification within the *T. cruzi* lineage, likely associated with ecological shifts and host colonization events.

The southern supercontinent hypothesis proposes that the *T. cruzi* lineage originated in association with South American marsupials, particularly opossums, around the time when South America separated from Gondwana [24]. This model suggests a long-term co-evolution between *T. cruzi* and terrestrial mammals of South American origin [24]. In contrast, the bat seeding hypothesis argues that the common ancestor of the *T. cruzi* clade, including *T. rangeli*, was originally a bat trypanosome. According to this model, trypanosomes diversified within chiropteran hosts and later underwent multiple independent host-switching events from bats to terrestrial mammals, one of which gave rise to modern *T. cruzi*. Maximum likelihood phylogenetic reconstructions support this hypothesis by clustering TcBat as basal to all other *T. cruzi* DTUs [25].

Further support for the bat seeding model comes from the detection of *T. cruzi* in the salivary glands of the hematophagous bat *Diaemus youngi*, suggesting that bats may participate actively in sylvatic transmission cycles. This opens the possibility that bats acted not only as ancestral hosts, but also as ecological bridges facilitating the parasite’s spread to other mammalian groups [26].

In summary, all these data indicate that *T. cruzi* emerged long before *Homo sapiens* (~200,000 years ago in Africa) [27]. By the time humans migrated to the New World around 30,000 years ago, they encountered a genetically diverse array of *T. cruzi* strains carried by arthropod vectors, in addition to infected terrestrial mammals. These early humans were thus exposed to *T. cruzi* infection first (and only much later to Chagas disease as a pathology) [25]. Initially, the humans who migrated into what is now South America were hunter-gatherers [28] participating in the sylvatic cycle of *T. cruzi* either by consuming meat from animals infected with the parasite (such as camelids or small rodents) or by inadvertently consuming the infected arthropod vectors themselves [29,30,31]. This is supported by evidence of very high *T. cruzi* infection rates in skeletal remains from various pre-Colombian civilizations of Latin America, such as Incas, Chinchorro, and others [29,30,31].

Notably, through PCR amplification of kinetoplastid DNA sequences, *T. cruzi* infection has been identified in ancient human remains, underscoring the long-standing role of oral transmission. Aufderheide et al. [32] examined 283 mummified bodies from southern Peru and northern Chile, finding a Chagas disease prevalence of approximately 41% over the past 9000 years. They suggested that oral transmission might have occurred through eating infected, undercooked meat (e.g., guinea pigs, *Cavia porcellus*). Likewise, TcI DNA was detected in Brazilian mummies dated to 4500–7000 years before present [33], and a mummy from the same region showing evidence of megacolon (a classic chronic Chagas pathology) was dated to around 560 ± 40 years A.P. (after present) [34].

PCR analyses of American ancient tissue samples have confirmed these findings. For example, tissues from six mummies (2000 BC–1400 AD) in the Archaeological Museum of San Pedro de Atacama (northern Chile) were tested, and four of the six (66.6%) had amplifiable *T. cruzi* DNA [28]. Similarly, a mummy ~1150 years BP from Lower Pecos, Texas (USA), showed evidence of *T. cruzi* infection, the oldest known infection in North America [35,36], and this individual also had pathological signs consistent with megacolon [30]. Analysis of coprolites (preserved feces) from that mummy suggests a remarkably diverse diet, including rodents, bats, and arthropods [30]. Such broad dietary habits raise the possibility that triatomines or even infected bats might have been eaten by these prehistoric populations of hunter-gatherers [35,36]. Additionally, an extremely high percentage (84%) of mummies from the Lower Pecos region exhibited dental abscesses, a condition that could have facilitated oral transmission of *T. cruzi* via open sores in the mouth [29].

Available evidence suggests that humans became involved in the *T. cruzi* transmission cycle on a larger scale once they settled into sedentary communities, domesticated animals, and began practicing agriculture (which in the Andean regions of South America occurred between 6000 and 8000 years ago) [22,31,37]. The presence of domesticated animals inside human dwellings, combined with the cultivation and storage of grains like wheat and maize, attracted rodent populations as well as triatomines. In human habitations, triatomine bugs found not only a variety of food sources but also shelter, which led to the establishment of a domestic *T. cruzi* transmission cycle.

Historical accounts suggest that oral transmission of Chagas disease may have occurred in prehistoric and early historic times. For example, chronicles from around the year 1330 describe an epidemic in Coyoacán (central Mexico). Following a disruption of the fish supply, inhabitants reportedly suffered high morbidity and mortality with symptoms including edema of the eyelids, face, and feet, and hemorrhagic diarrhea. Many people died, mainly the young and the elderly. This clinical picture may suggest a possible case of foodborne outbreak of Chagas disease, possibly acquired after hunting and consuming infected opossums (*Didelphis* spp.), which are natural reservoirs of *T. cruzi* [38].

In the modern scientific literature, *T. cruzi* human oral infection was observed first in animal models in 1913 [39]. Subsequently, experimental infections in mice using blood contaminated with trypomastigotes, urine, and feces from triatomines infected with *T. cruzi* confirmed this potential mode of transmission [40]. The first human cases of orally acquired Chagas disease were reported by Silva et al. in 1968 [9,41]. Subsequently, Yaeger in 1971 documented the transmission of *T. cruzi* to opossums via the oral route, suggesting that wild animals could become infected by ingesting food or water contaminated with metacyclic trypomastigotes (e.g., via anal gland secretions of infected opossums) [42]. In fact, some reported human oral infection cases have been attributed to contact with opossum secretions [42].

Oral transmission was initially studied in animal reservoirs and wild vectors by Barretto et al. (in 1978) [43]. It is considered the ancestral mode of parasite transmission, as it is the most common form of infection between animals and vectors and has allowed *T. cruzi* to be maintained in nature over millennia [44]. In the earliest stages, Chagas disease was an endemo-enzootic infection, with *T. cruzi* cycling in wild environments largely through oral transmission, when different animals ingested infected vectors or through carnivory in predator–prey interactions [11,45].

All this evidence together suggests that oral transmission was especially relevant in the early peopling and settlement of the Americas by indigenous cultures.

## 3. The Current Role of the Oral Route in Human *T. cruzi* Infection

**Figure 2:**
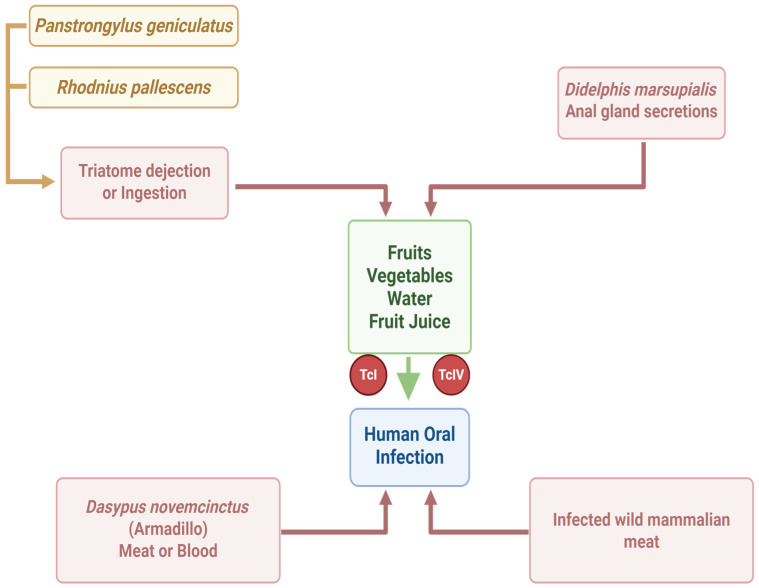
Concept map of human oral infection by *Trypanosoma cruzi* (created in https://BioRender.com).

Currently, oral transmission of *T. cruzi* occurs as a result of ingesting food and beverages contaminated with the parasite. This typically happens either by blending triatomine insects (or their feces) into food products. For example, contamination can occur in fruit juices (such as açaí or sugar cane juice) when triatomines or their droppings are ground up with the fruit, or via tainted foods. A concept map of oral transmission is shown in Figure 2.

Outbreaks of orally acquired Chagas disease have been described in several other South American countries as well, including Venezuela, Colombia, Bolivia, Argentina, French Guiana, and Ecuador [12,46,47,48,49,50,51,52]. These outbreaks have been associated with consuming a variety of contaminated foods and drinks, such as wild game meat (e.g., armadillo), fruits or vegetables, sugar cane juice (“guarapo”), açaí palm fruit pulp, guava juice, bacaba (palm fruit) juice, babaçu coconut, and even wine made from palm fruit [12,13,53,54,55]. From 1965 to 2000, about 50% of acute Chagas cases in the Amazon region were attributed to oral transmission [12,13], and between 2000 and 2010, this proportion reached 70%. Venezuela experienced the largest documented outbreaks: two urban school-based outbreaks affecting 103 and 88 people, respectively (including both children and adults) [10,18]. The total number of cases and outbreaks of oral *T. cruzi* infection described to date, indicating, where possible, the source of infection and the DTU involved, is shown in Table 1.

Importantly, orally acquired infections tend to be more severe. The mortality rate in orally infected patients ranges from 0 up to 44%, which is significantly higher than the <5–10% mortality seen in vector-transmitted (bite-associated) acute Chagas cases [48]. In summary, currently, the oral route is the most frequent and significant mode of *T. cruzi* transmission in many areas [9]. In some regions of the Americas, oral transmission of *T. cruzi* has not yet been reported, but the risk of such outbreaks remains real. Human cases of oral infection could occur mainly in two scenarios:

1. Tourist or non-local populations visiting tropical areas of countries like Brazil, Colombia, or Venezuela, who may consume local foods, juices, or fruit pulps that are contaminated with infected triatomine feces, or who eat raw/undercooked “exotic” meats (such as armadillo).
2. Communities that consume meat from free-ranging domestic or wild animals (e.g., rabbits, hares, or goats) in areas where wild triatomines are present.

For example, in Chile, the consumption of undercooked meat from goats or rabbits could pose an oral transmission risk. Indeed, infections in Chilean rabbits and goats have been reported [98,99]. Additionally, climate change may expand the range of wild triatomine vectors like *Mepraia gajardoi* (Reduviidae, Triatominae) and *Mepraia spinolai* (Reduviidae, Triatominae) (the main wild vectors in Chile) [100]. On the other hand, various traditionally domestic animals such as pigs [101], sheep, and horses have also been found infected with *T. cruzi* in different places in Latin America [102,103].

All these factors make oral transmission of *T. cruzi,* particularly through carnivory (eating infected meat), an emerging challenge for Chagas disease control. It is necessary to deepen our understanding of the biological and molecular determinants that enable BT or tissue culture-derived trypomastigotes (TCT) to establish infection via the oral route, using appropriate animal models for both acute and chronic infection.

Thus, in all cases, a critical event is preventing infection, where laboratory studies have provided useful information in this direction. Protective measures include covering and sealing food containers to prevent contact with insects, pasteurizing fruit juices (as is now mandated for açaí pulp in some regions), and freezing wild game meat before consumption to kill parasites. This is particularly relevant, considering that experimental studies have shown *T. cruzi* survival and virulence in açaí pulp are unaffected by prior incubation at room temperature for 24 h, at 4 °C for 144 h, or at –20 °C for 26 h. These results indicate that *T. cruzi* can survive and remain virulent in açaí pulp under various conditions, and that cooling or brief freezing are not adequate methods for preventing *T. cruzi* infection via oral consumption [104]. On the other hand, heating açaí pulp above 43 °C for 20 min can prevent foodborne acute Chagas disease [105]. In vitro experiments have demonstrated a loss of *T. cruzi* trypomastigote viability, which was only complete after 120 h at –20 °C and 144 h at +2 °C, whereas parasites remained alive after 168 h at −80 °C [106]. On the other hand, the parasite can survive in sugarcane juice for 4 to 12 h [106]. A summary of the main preventive measures useful for people living in endemic areas with vector transmission and for tourists is shown in Table 2.

## 4. Clinical Aspects of Orally Acquired Chagas Disease

The clinical course of Chagas disease is commonly divided into acute, indeterminate (asymptomatic), and chronic phases [107]. If symptoms develop during acute infection, they are usually mild and nonspecific. After the acute phase, patients enter an indeterminate (asymptomatic) phase, during which no clinical symptoms are present (although infection can be serologically detected for life). Approximately 30% of infected individuals eventually progress from the indeterminate phase to the chronic phase of Chagas disease. The most significant clinical manifestation of chronic Chagas is cardiomyopathy, which develops in about 20–30% of infected people. Chagas can also affect the gastrointestinal tract (GI) and the nervous system; the most common GI manifestations are megaesophagus and megacolon [17]. The drugs currently used to treat Chagas disease are nifurtimox and benznidazole. These are considered effective for treating acute and early chronic infection in children under 15 years of age. However, their efficacy and therapeutic value in chronic infection is controversial [108].

Unlike vector-borne acute Chagas infections (which are often asymptomatic or very mild), acute Chagas disease acquired through the oral route is usually symptomatic and presents with more severe manifestations, mainly affecting children and young adults (<18 years old). However, the clinical manifestations can vary greatly from patient to patient [18], likely depending on the size of the inoculum and the host’s immune response. After an incubation period of roughly 3 to 22 days following ingestion of *T. cruzi*, the first sign is often an unexplained, persistent high fever. This is typically accompanied by symptoms such as headache, profound weakness, and edema of the face and extremities. Acute myocarditis evidenced by arrhythmias, pericardial effusion, and cardiomegaly is another prominent feature of orally acquired infection [107]. Mortality in outbreaks of orally transmitted Chagas disease can reach as high as one-third of those infected (though it varies, reported between 5% and 33%) [109,110]. Higher acute mortality has been linked to several factors: *T. cruzi* strain involved [111], the patient’s age, the parasite load in the infected host, and delays in diagnosis and treatment [13]. Similarly, the infection appears to be more severe in children, the elderly, and immunocompromised individuals [6,13].

**Figure 3:**
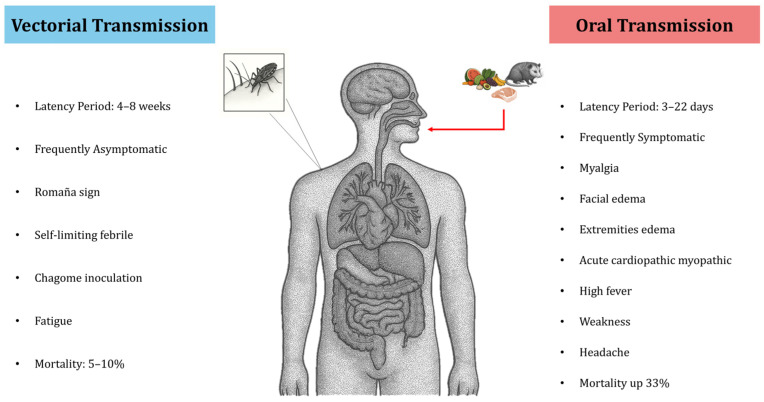
Main differences in the clinical presentation of Chagas disease according to the mechanism of infection.

A study by Dos Santos et al. [112] examined changes in liver function and coagulation factors in 102 patients with acute Chagas disease acquired orally in the Brazilian Amazon. The most frequent symptoms were fever (98% of cases), asthenia (83.3%), edema of the face and limbs (80.4%), headache (74.5%), and myalgia (72.5%). Levels of liver enzyme alanine aminotransferase (ALT) and aspartate aminotransferase (AST) were higher in 30 of these acute patients compared to uninfected controls. Moreover, patients showed elevated plasma levels of activated protein C and reduced levels of factor VII (a clotting factor). The study concluded that oral *T. cruzi* infection is associated with disseminated intravascular coagulation and liver function impairment [112]. Thus, the clinical presentation of acute Chagas disease differs markedly depending on the route of infection. Vector- borne infection tends to be mild or asymptomatic, whereas oral infection often leads to severe, symptomatic disease. These differences are illustrated in Figure 3.

### Clinical and Laboratory Diagnostics

Laboratory diagnosis presents some difficulties, such as the fact that no single technique is capable of detecting 100% of cases. In addition, in some cases, it is necessary to take more than one blood sample. Furthermore, reference laboratories are generally located far from the site of the outbreak, which delays the arrival of samples and, therefore, diagnosis. However, the detection of parasites using direct methods, such as fresh blood observation and microhematocrit, is a rapid method that can be performed without sophisticated equipment and has good sensitivity in the acute phase of infection [113].

Serological diagnosis of acute infection cases due to contaminated food consumption has traditionally been based on the detection of IgG and IgM using ELISA and immunofluorescence. However, the most appropriate procedure is to request three blood samples: one in a tube without anticoagulant for serology; one collected in a tube with heparin for culture, microhematocrit, and inoculation in animals [113,114]; and a third sample collected in a tube with ethylenediaminetetraacetic acid for PCR [115].

However, sometimes, the clinical manifestations may be nonspecific, which makes diagnosis a real challenge for clinicians, given that oral acute Chagas disease can be confused with other acute febrile illnesses, such as leptospirosis, visceral leishmaniasis, malaria, enteric fever, rickettsioses, and some arboviruses [116]. Then, the following criteria must be used in confirming acute Chagas disease cases: (1) exposure to the probable source of contamination; (2) manifestation or absence of symptoms; and (3) confirmation of laboratory diagnosis by parasitological tests, among them thick blood smear, hemoculture, xenodiagnoses, PCR, and serological tests [83,115].

## 5. Oral Infection by *T. cruzi* in Animal Models

In laboratory animal models, oral *T. cruzi* infection can be established either by direct inoculation into the oral cavity (oral infection, OI) or by gastric intubation/gavage (gastric infection, GI). These two methods represent slightly different routes of entry, yet both produce clear parasitemia and heart tissue infection during the acute phase. Barreto-de-Albuquerque et al. [21] provided conclusive evidence that the initial site of parasite entry dramatically affects the host’s immune response and disease outcome. In their study, mice infected via OI showed higher parasitemia levels and greater mortality than mice infected via GI, even when the same parasite strain and dose were used. Interestingly, heart inflammation was more extensive in GI-infected mice, whereas liver lesions were more severe in OI-infected animals, accompanied by higher serum ALT and AST levels in the OI group [21]. This indicates that the entry route alters the tissue tropism and pathology of infection.

Another study compared oral inoculation to intraperitoneal (IP) inoculation in mice, using different DTUs and inoculum sizes. Overall, mice infected orally showed lower parasitemia, infectivity, and mortality rates than those infected via the IP route, regardless of the parasite strain (DTU) or dose [117]. Within this study, mice infected with a TcII strain had higher parasitemia and mortality rates than those infected with a TcI strain. A larger volume of inoculum caused a higher infection rate when administered orally. BT challenges were more virulent than MT challenges for both oral and IP routes. However, even for a virulent BT strain, orally inoculated mice had lower parasitemia and mortality than mice inoculated IP. In contrast, oral inoculation with culture-derived MTs produced infection levels similar to IP, and those MT-inoculated mice showed more histopathological changes than BT-inoculated mice (for both routes) [117]. These findings imply that *T. cruzi* virulence and pathology depend on a combination of the parasite form, the dose, and the route of entry.

Silva-dos-Santos et al. [118] used bioluminescence imaging and quantitative PCR to study oral *T. cruzi* infection in mice. They infected mice with a bioluminescent strain of *T. cruzi* (Dm28c-luc, a TcI strain) via the oral route. In vivo imaging showed that the nasomaxillary region (around the mouth and nose) is the initial site of parasite invasion. By 7–21 days post-infection, the bioluminescence signal intensified in the thorax, abdomen, and genital area, reflecting dissemination of the parasite to different tissues. Similarly, using a fluorescent strain (Dm28c-GFP), nests of *T. cruzi* amastigotes were detected in the nasal cavity by day 6 post-infection. Quantitative PCR at days 7 and 21 post-infection showed that the parasite was predominantly found in the nasal cavity and had expanded there. These results clearly demonstrate that the oral cavity and adjacent structures (like the nasal passages) are the primary target regions in oral *T. cruzi* infection, leading to early parasite multiplication in the nasal mucosa.

A characteristic feature of oral-route *T. cruzi* infection is prominent facial and hind limb edema. In some cases, hemorrhagic manifestations and risk of thromboembolism are also observed. In one investigation, BALB/c mice were orally infected with MT of the Tulahuén strain, and researchers analyzed the cytokine response and hemostatic changes during acute infection. Orally infected mice, compared to uninfected controls, showed markedly elevated serum levels of proinflammatory cytokines (TNF-α, IFN-γ, IL-6), as well as leukocytosis and thrombocytopenia. These hematologic changes were accompanied by prolonged activated partial thromboplastin time, consumption of factor VIII, and increased D-dimer levels—a pattern indicative of disseminated intravascular coagulation [119].

Another study evaluated the digestive tract pathology in OI mice with the *T. cruzi* Berenice-78 (Be-78) strain, compared to mice infected via the intraperitoneal route. Both groups exhibited a similar peak in parasitemia, but OI mice had a higher mortality rate than IP-infected mice. When examining immune cell populations, the OI group showed a prolonged presence of CD4^+^ T-lymphocytes (lasting longer than in the IP group). Histopathologically, OI mice had greater parasite loads and inflammatory infiltrates in the stomach, duodenum, and colon at 28 days post-infection. These data suggest that OI induces a different profile of parasitological and immune responses compared to IP infection, with oral infection being more virulent and producing greater tissue parasitism in gastric organs during acute *T. cruzi* infection [120].

When BALB/c mice were infected with the Colombian strain (TcI) via different routes (IP, OI, GI, ocular, and cutaneous), and using either culture-derived MT or BT, distinct patterns were observed [20]. Parasitemia remained intermittent and low in mice inoculated with MTs (for all routes), whereas mice inoculated with BTs developed high parasitemia. A muscle tropism was noted in oral or GI infections with BT forms, whereas MT infections led to a wider distribution of the parasite in various tissues. Interestingly, tissue inflammation was more intense in oral or GI infections with BT. Mice inoculated with BT via the GI route had a similar IFN-γ level, but lower IL-10 levels compared to mice infected with MTs via GI. Additionally, TNF-α levels were higher in BT-infected mice, which could explain the more severe heart inflammation seen in those animals [20].

It has also been reported that in mice, oral *T. cruzi* infection leads to diffuse parasitism in bone marrow cells, targeting perivascular and intravascular regions and areas close to bone. Extramedullary hematopoiesis observed in the spleen during oral infection may be a compensatory mechanism to maintain blood cell production during the acute phase of *T. cruzi* infection [121].

**Figure 4:**
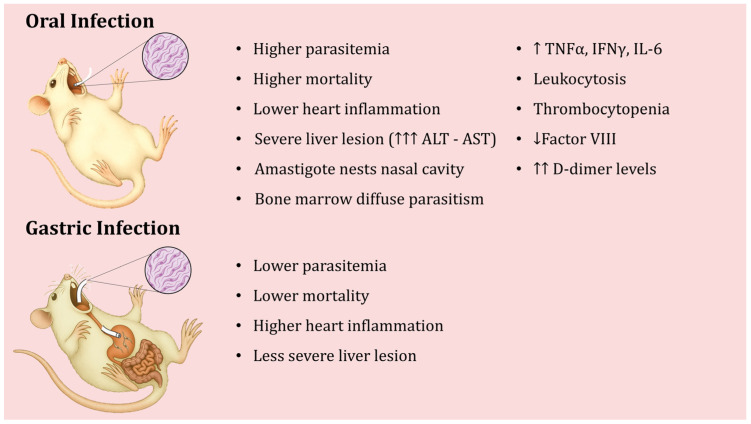
Differences in the course of experimental oral infection with *Trypanosoma cruzi* depending on whether inoculation is oral or gastric.

The basis of *T. cruzi* virulence via the oral route has been investigated using a particularly virulent isolate. One study analyzed a *T. cruzi* isolate called “SC” obtained from a patient with severe acute Chagas disease acquired orally, using a mouse model. It was observed that when mice were inoculated orally with the metacyclic forms of the SC isolate (which express high levels of the surface glycoprotein gp90), the mice developed high parasitemia and high mortality, despite the SC isolate showing reduced infectivity in vitro [122]. Upon recovering parasites from the mouse stomach 1 h after oral inoculation, the gp90 molecule on the parasites was found to be completely degraded, and the parasite’s ability to invade cultured cells (HeLa and Caco-2) was markedly increased [122]. In contrast, the parasite’s gp82 surface molecule was more resistant to digestive enzymes in gastric juice, and the presence of gastric mucin further enhanced host cell invasion by the MT of the SC strain. Notably, reference strains CL and G, which express gp90 isoforms that are resistant to gastric degradation, did not show changes in infectivity after exposure to gastric juice. Taken together, these findings suggest that an exacerbation of *T. cruzi* infectivity after interaction with host stomach components may underlie the severity of acute Chagas disease in human oral outbreaks [122]. In the case of the SC isolate, the loss of gp90, due to its digestion, presumably allowed the parasites to invade host tissues more effectively, contributing to severe disease. The main characteristics of the experimental murine infection, depending on whether it occurs orally or gastric, are shown in Figure 4.

## 6. Animals as Reservoirs and Spreading Factors

Mathematical models have shown that oral transmission among domestic animals can substantially contribute to the spread of Chagas disease [123]. Oral transmission provides an alternative route to vector bites for infecting domestic mammals, which are key reservoir hosts in the infection cycle. This means that even if triatomine bugs do not frequently bite certain domestic animals, those animals might still get infected (and subsequently infect vectors) by orally ingesting insects or contaminated food. Such a dynamic can lead to high infection rates in domestic mammals and ultimately maintain high infection pressure in humans [122].

In nature, over 180 species across 25 mammalian families have been described as potential reservoirs of *T. cruzi* [9]. In documented oral transmission outbreaks, about 70% of implicated reservoir hosts are various species of marsupials didelphid (opossums), such as *Didelphis marsupialis*, *D. aurita*, and *D. albiventris*, as well as other opossum-related species like *Marmosa spp., Monodelphis domestica*, and *Caluromys lanatus*. These animals are often synanthropic and can act as both reservoirs and, indirectly, vectors (because predators or scavengers may ingest them). The next most common reservoirs, each accounting for ~10% in those outbreaks, are dogs (*Canis lupus* familiaris), black rats (*Rattus rattus*), and certain wild rodents such as *Trichomys laurentius* (São Lourenço punaré) and *Cavia aperea* (Brazilian guinea pig). Dogs, in particular, were identified as domestic reservoir hosts as early as 1969 [44]. Dogs not only serve as a blood source for triatomines but can also ingest insects or food contaminated with triatomine feces, thus participating in both vectorial and oral transmission routes. Because dogs live in such close contact with people, they are excellent sentinels for parasite circulation in domestic environments [124]. In urban settings, rats are often the main urban reservoir of *T. cruzi*, followed by dogs and cats, in maintaining a peridomestic cycle [125]. Other naturally infected reservoirs include various wild rodents. In Venezuela, for example, species like *Dasyprocta agouti* (agouti) and *Trichomys apereoides* have been found infected [125]. In Brazil and other regions, wild rodents such as *Echimys dasythrix* and species of Akodon have also been noted in the wild cycle [126].

In Chile, the epidemiological scenario appears slightly different due to the absence or low presence of some typical vector species in domestic settings. However, very high infection rates have been observed in wild rodents there [126]. This could be explained by oral transmission in the wild: rodents might be ingesting infected triatomines (*Mepraia* species) [127] or food contaminated with triatomine feces. *Mepraia spinolai* (Reduviidae, Triatominae), a wild triatomine in Chile, has infection rates ranging from ~40% up to 76% in some studies [128,129]. PCR surveys have found *T. cruzi* DNA in 25–41% of tested individuals of the wild rodent *Phyllotis darwini*, 13–70% of *Octodon degus* (degu rodents), and ~27% of *Rattus rattus* in certain sylvatic areas of Chile [130]. Separately, a study in rural Chilean homes found 83.1% of captured rodents (commensal rodents near houses) were infected; among these, *P. darwini* had 100% infection, *R. rattus* 83.6%, *Mus musculus* 83.3%, and *O. degus* 50% [127]. There is even evidence of *T. cruzi* infecting reptiles; for instance, in one study from Chile, *T. cruzi* DNA was detected in 11 of 13 *Microlophus* (lizard) blood samples and in all tested individuals of certain lizard species (18 *Liolaemus platei* “Plate’s lizards”, 3 *Liolaemus nigroviridis* “Dark lizards”, and 10 *Homonota penai* “marked geckos”) [131]. Such findings lead to the hypothesis that carnivorous or omnivorous animals (like cats or pigs) could become infected by eating smaller infected animals (e.g., rodents or lizards). For example, cats or feral pigs might prey on or scavenge infected rodents, thus acquiring *T. cruzi* orally.

In Chile’s central region, PCR-based studies have shown *T. cruzi* infection in 17–57% of dogs [130,132,133], and about 11% of dogs in the north were seropositive [133]. In central Chile, *Mepraia spinolai* is the prevalent vector, but this insect has a long defecation delay after biting (on average ~8 min) [134], meaning it often defecates after leaving the host, reducing the efficiency of classical transmission. This raises the question: how are so many dogs and wild rodents infected in those areas? A plausible explanation is oral transmission—for instance, rodents (or other animals) might ingest insects or food contaminated with *M. spinolai* feces. This would support a significant role for the oral route of infection in such rural or semi-wild environments, even in countries like Chile where no human oral cases have been officially reported yet.

## 7. What We Know About *T. cruzi* DTUs and Mechanisms Involved in Oral Infections

In northern South America and the Amazon region, where most oral Chagas outbreaks have occurred, the predominant *T. cruzi* genetic group is DTU TcI [135,136,137]. TcI was the genotype identified in parasites isolated from patients in orally transmitted outbreaks in Venezuela and Colombia [50,126]. TcI also predominates in Mexico [138,139]. *T. cruzi* strains used in oral infection research to date have included isolates from Guatemala, Venezuela, and various regions of Brazil [82,122,140,141]. Interestingly, two orally transmitted outbreaks in northern Brazil involved *T. cruzi* strains of DTUs TcI and TcIV [82].

According to Barbosa et al. [142], metacyclic forms of Mexican *T. cruzi* strains (TcI) are poorly infectious by the oral route and invade cultured cells inefficiently. Consistently, those Mexican TcI strains caused either undetectable or very low parasitemia when mice were inoculated orally. This outcome is similar to what has been observed with other Latin American TcI strains in oral models [82]. It contrasts with findings for some TcII and TcVI strains, which have produced patent parasitemia in mice and efficiently invaded host cells in vitro via the oral route [117,141,143,144]. Despite the genetic diversity of *T. cruzi*, a common theme from these studies is the reduced oral infectivity of TcI (and, to a lesser extent, TcIV) strains. In other words, MT of TcI parasites seem less capable of migrating through the gastric mucus, invading target epithelial cells in the stomach, and establishing infection, compared to strains from DTUs like TcII or TcVI [117,141,143,145].

Furthermore, a study by Lewis et al. [145] using highly sensitive bioluminescence imaging and quantitative histopathology shed light on oral infections in mice with another perspective. They found that both MT and BT could establish infection via the oral cavity, but only MT forms led to consistent infections when delivered directly to the stomach by gavage. In their experiments, they used MT and BT forms from the CL Brener strain (TcVI), and noted that while both forms were orally infective, the metacyclic forms were more efficient at establishing infection through the gastric route.

Most existing knowledge about oral *T. cruzi* infection mechanisms comes from studies on MT [146]. In vitro-generated MT forms (analogous to the stage transmitted by insect vectors) have been studied to identify factors important for oral infection. One key surface molecule is gp82, which plays an essential role in host cell invasion and the establishment of infection via the oral route. Gp82 has the ability to bind gastric mucin and induce host cell signaling cascades that result in actin cytoskeleton rearrangement and lysosome exocytosis events that facilitate parasite internalization [147]. Gp82 is also relatively resistant to pepsin digestion at acidic pH, allowing it to retain its function in the stomach environment. Conversely, another surface molecule, gp90, is a stage-specific glycoprotein that negatively *T. cruzi* strains expressing high levels of gp90 typically invade host cells poorly in vitro. However, their infectivity by the oral route can vary because different gp90 isoforms have different susceptibilities to pepsin. Parasites with gp90 that are easily degraded by gastric juice become highly invasive once that inhibition is lifted [140]. Parasites expressing gp90 isoforms resistant to digestion do not gain such an advantage in the stomach [140].

An example of DTU-related differences involves a bat-associated strain. MTs from the BAT strain (a strain isolated from bats) were shown to migrate through an artificial gastric mucin layer and infect HeLa cells in vitro. However, MTs of the BAT strain were less effective than those of CL or G strains at infecting mice via oral or IP routes [148]. Meanwhile, multiple outbreaks of acute orally acquired Chagas in the Brazilian Amazon have been linked to TcIV strains, which appear quite infective by the oral route in mice. In one comparative study, four TcIV strains from Amazonian outbreaks were tested in mice: orally inoculated mice had higher peak parasitemia, higher positivity rates by PCR, and higher parasite loads in tissues than mice inoculated with the same strains via IP injection. This indicates a higher intensity of infection when these strains are acquired orally (perhaps because the oral route targets different initial tissues or evades some immune responses) [144].

**Figure 5:**
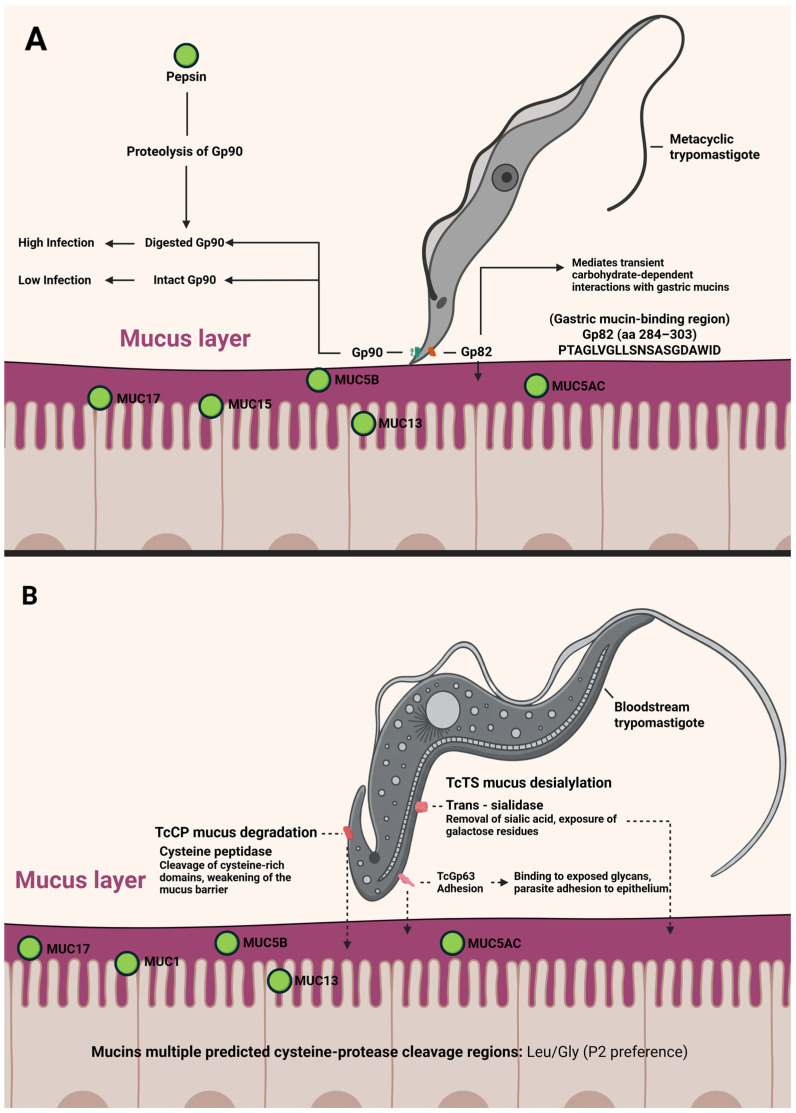
A model of metacyclic trypomastigote (**A**) and tissue culture derived trypomastigote or blood trypomastigote (**B**) interaction with the mammalian gastric mucosa. In all cases, the solid line corresponds to experimentally verified events, while the dotted line corresponds to events based on indirect evidence or bioinformatic analysis. (Created in https://BioRender.com).

While the molecular basis of MT interactions with the gastric environment is fairly well characterized [146], much less is known about BT in oral infection. Earlier studies suggested that BTs are inefficient at causing infection via the oral route in mice [149] and some even proposed that carnivory might not be a significant transmission mode [150]. Nevertheless, many observations, old and new, suggest that carnivory (predator–prey transmission) can play a central role in *T. cruzi* spread, especially in wild cycles. Predators consuming infected prey could become infected through their mucosal membranes while chewing or digesting the meat [45,55]. Only recently have there been experimental data to directly support this route. In a recent paper, Torres et al. [151] have reported that consuming meat from *T. cruzi*-infected animals in the acute phase can indeed transmit the infection to other animals, providing the first direct experimental evidence that carnivory is a viable transmission mechanism for Chagas disease. In these experiments, trypomastigotes and even intracellular amastigotes from a TcI *T. cruzi* clone (H510 C8C3) were capable of infecting mice when administered orally (either by feeding infected tissue to mice or by gavage) [151]. In all cases, 60–100% of mice that consumed meat from acutely infected donors (or were fed trypomastigote/amastigote suspensions) developed high parasitemia, and up to 80% succumbed to infection [151]. These observations also shed light on why bloodstream forms are less infective orally: according to these authors, trypomastigote motility in the presence of gastric mucin was reduced by ~30%. When both mucin and pepsin (at pH 3.5) were present (simulating stomach conditions), only about 6% of trypomastigotes managed to migrate across a filter in vitro. Similarly, the ability of TCT to infect human gastric epithelial cells (AGS cell line) in the presence of mucin was reduced by ~20%. The available evidence suggests that MTs belonging to the DTU TcI would be less infectious than other DTUs. However, this is the first reported evidence of oral infectivity of TCT from this DTU and therefore raises some questions. Are TcI TCT more infective than their MT counterparts? Are there differences in the infectivity of different strains belonging to TcI? Only experimental trials can answer this question, but it has been suggested that TcI has shown great genetic diversity, indicating plausible subdivisions needed for this group [152]. This report also identified potential parasite proteins involved in overcoming these barriers: an in vitro adhesion assay showed that cruzipain (Czp), a major cysteine protease of *T. cruzi*, can bind to AGS cells. Additionally, LC-MS/MS proteomic analysis suggested that *T. cruzi* trans-sialidase (TS), other cysteine proteinases (CPs), and gp63 metalloprotease could all interact with proteins of the human stomach mucosa, implicating them in attachment or invasion processes. Notably, several human gastric mucins have cleavage sites for cysteine proteases like cruzipain [151]. All these data allowed us to propose a model of interaction between orally introduced *T. cruzi* (especially TCT) and the gastric mucosa, and compare this with what we know about the interaction of MTs with the gastric mucosa. A model of trypomastigote interaction with the gastric mucosa is shown in Figure 5.

In summary, consuming meat from animals infected in the acute phase can transmit *T. cruzi* infection orally. However, it remains to be investigated whether oral transmission is possible from animals in the indeterminate or chronic phase of infection. It is conceivable that in later stages, the success of oral transmission would depend on the parasite load in specific organs/tissues and on the parasite’s genotype (DTU), which might determine its ability to resist stomach acidity, penetrate mucus, and invade gastric cells. Thus, it is reasonable to hypothesize that the particular *T. cruzi* DTUs involved in food contamination could be critical determinants of oral transmission success. Indeed, earlier studies by Hoft et al. [149] noted poor oral infectivity of blood trypomastigotes from the Tulahuén strain (TcVI), and Cortéz et al. [143] observed that trypomastigotes of the Y strain (TcII) were also poorly infective orally. It is also likely that the intrinsic virulence of each strain or isolate (for example, higher expression of virulence factors such as cruzipain, trans-sialidases, and gp63) plays a role [153]. Furthermore, recent research suggests that the upregulation of certain metabolic and biosynthetic pathways in the parasite might contribute to virulence [154]. Finally, the genetic background of the host is another important factor. A parasite strain highly infective in one host species might be much less so in another species.

It is also necessary to consider that the infectious inocula used in laboratory models may differ from those that a host might receive in nature. For example, in food contaminated by the presence of a complete triatomine, all existing trypomastigotes are present in its intestinal contents, which in *T. infestans* can reach 684,000 trypomastigotes [155], theoretically enough to infect more than a hundred people. However, in the fecal matter of a triatomine, it is estimated to be between 3000 and 4000 metacyclic trypomastigotes per microliter of feces [155]. Although there is insufficient information regarding the parasitemia that wild animals could develop, one report mentions high parasite levels in wild pigs [156]. Studies with goats experimentally infected with *T. cruzi* showed peak parasitemia levels of 1.43 × 10^6^ parasites by mL^−1^ on day 12 post-infection. However, the parasites were not detected 22 days post-infection [157]. Nevertheless, some of these animals tested positive in xenodiagnosis seven months after inoculation. Similarly, goats naturally infected with *T. cruzi* have shown low levels of parasitemia, suggestive of chronic forms of *T. cruzi* infection [157].

In summary, the findings reported by Torres et al. [151], although interesting, should be taken with caution, as the conditions of the experimental models may not always reflect what actually occurs in nature. On the one hand, genetic background could be a determining factor. Pioneering work by Pizzi showed that not all mouse strains have the same susceptibility to *T.cruzi* infection [158]. Therefore, there could be species in nature that are refractory to infection or that, even in the acute phase, have low parasitemia. It is known that some strains of *T. cruzi* such as G [159] or C8C3*lvir* [154] in laboratory models give subpatent parasitemia. On the other hand, studies in wild animals have shown seropositivity for *T. cruzi* in the presence of negative blood culture and PCR results. These cryptic infections are indicative of a subclinical infection at the time of blood collection. However, given that the transmission systems of multi-host parasites are dynamic over time, animals without parasitemia could present high parasitemia during periods of transient immunosuppression due to malnutrition, concurrent infections, pregnancy, and lactation [160].

## 8. Concluding Remarks and Future Perspectives

Acute Chagas disease resulting from the consumption of food or meat contaminated with *T. cruzi* from infected animals has emerged as the primary mode of transmission in areas of Latin America where wild or peridomestic triatomine vectors persist. This epidemiological shift presents new challenges in multiple domains.

Implementation of Control and Prevention Strategies: The encroachment of triatomine vectors and *T. cruzi* reservoir hosts into urban and periurban areas increases the risk of oral transmission. Control strategies must therefore adapt to these changing conditions. This includes not only traditional vector control measures (insecticide spraying and housing improvements to exclude insect vectors) but also enhanced food safety interventions. Reducing poverty and improving housing in rural and peripheral urban regions will decrease human contact with vectors. Education is crucial: communities must be informed about the risk of consuming food contaminated with triatomine feces or infected meat. Only when people recognize these risks will they adopt proper food hygiene practices. In most Brazilian states and countries where oral transmission has been reported, with the exception of açaí treatment, these prevention programs do not exist.

For widely distributed commercial products like açaí pulp, routine screening for *T. cruzi* contamination (for example, by PCR) should be considered. Tourists visiting endemic areas should also be cautioned against consuming uncooked foods or beverages (such as fresh palm juice) that might be contaminated. Such public health messaging, including school-based programs, could help prevent future outbreaks. Therefore, safe food production programs must be urgently implemented, aligned with improved diagnostic and therapeutic tools to combat foodborne Chagas [55].

Diagnosis and Treatment of Orally Acquired Chagas Disease: Outbreaks of oral *T. cruzi* transmission often involve many patients with fulminant acute disease, which can overwhelm local health services. Early diagnosis and treatment are vital, as the prognosis improves significantly with prompt antiparasitic therapy during the acute infection.

Medical personnel in endemic and at-risk areas must be trained to recognize the signs of acute Chagas disease, especially in scenarios suggestive of foodborne transmission (for example, multiple patients presenting with fever and edema after sharing a common meal).

Strengthening laboratory diagnostic capacity for acute Chagas (serology, PCR, microscopy) is also important. Furthermore, continuous medical education programs should incorporate information on orally transmitted Chagas disease so that physicians, laboratory technicians, and public health workers can respond quickly and effectively when an outbreak is suspected.

Advancing Knowledge of *T. cruzi* Biology in Oral Transmission: Despite recent progress, there remain many unknowns about how *T. cruzi* establishes infection via the oral route. Key questions include whether the success of oral infection depends on the parasite’s life stage or genotype. Our experimental evidence showed that a clone of *T. cruzi* (TcI, clone C8C3*hvir*) was able to infect BALB/c mice via oral inoculation (either by gavage or by feeding the mice infected meat). However, it is not yet known whether all *T. cruzi* discrete typing units (DTUs) are equally capable of oral infectivity, or if some strains are naturally attenuated in their ability to establish infection via this route.

It is also unclear whether meat from animals in the chronic or indeterminate phase of infection (which carry lower parasite loads) can cause oral infections notably; all documented oral Chagas outbreaks in humans have been traced to acutely infected sources (e.g., freshly infected game animals or heavily infected vector droppings). Similarly, it remains unknown whether all common laboratory animals (and by extension, all potential reservoir host species) are equally susceptible to oral infection by all *T. cruzi* strains, or whether certain host–parasite combinations are less permissive to infection. Another critical area of inquiry is the mechanism of mucosal traversal: what parasite factors enable *T. cruzi* to survive and penetrate the host’s gastric environment?

Oral transmission of Chagas disease has thus shifted from being considered a rare or unusual event to being recognized as a significant epidemiological route, especially in certain regions. Addressing this form of transmission will require interdisciplinary efforts—from improving food safety and public awareness to deepening our scientific understanding of how *T. cruzi* behaves in the host digestive system. Future research and public health initiatives must confront these challenges in order to reduce the incidence of orally acquired Chagas disease.

## Figures and Tables

**Table 1:** Reported oral Chagas disease outbreaks in South América.

Outbreaks Places	Year of Occurrence	Cases (N_0_)	Deaths (No.%)	95% CI	DTU	Vehicle	References
**Bolivia**							
Guayaramerín, Beni	2010	14	0 (0%)	[0.00–23.16]	NR	Majo	[56]
**Brazil**							
Río Grande Do Norte	1965	17	6 (35.2%)	[14.2–61.67]	NR	NR	[57]
Belém, Pará	1968	3	1 (33.3%)	[0.84–90.57]	NR	NR	[58]
Macapá, Ap	1984	8	0 (0%)	[0.00–36.94]	NR	NR	[59]
Paraíba	1986	26	1 (3.8%)	[0.10–19.64]	NR	Cane sugar juice	[60]
Río do Branco, Acre	1993	3	3 (100%)	[29.4–100.0]	NR	Açaí	[61]
Mazagao, Ap	1996	17	0 (0%)	[0.00–19.51]	Tc I; TcIIa/TcIIb	Açaí	[62]
Abaetetuba, Pará	1998	13	0 (0%)	[0.00–24.71]	NR	NR	[63]
Cametá, Pará	1999	3	2 (66.6%)	[9.43–99.16]	NR	NR	[64]
Pará, Macapá, Amapá	1990–2003	20	3 (20%)	[3.21–37.89]	NR	NR	[65]
Tefé, Amazonas	2004	1	0 (0%)	[0.00–97.50]	NR	Açaí	[66]
Pará, Amapá, Maranhao	1998–2005	183	13 (7.1%)	[3.84–11.84]	TcI	NR	[67]
Tefé, Amazonas	2005	9	0 (0%)	[0.00–33.63]	NR	Açaí	[68]
Santa Catarina	2005	24	3 (12.5%)	[2.66–32.36]	TcI; TcII	Cane sugar juice	[69]
Bahía	2006	7	2 (28.5%)	[3.67–70.96]	NR	Water?	[49]
Pará	2006	11	0 (0%)	[0.00–28.49]	NR	Açaí	[70]
Ceará	2006	8	0 (0%)	[0.00–36.94]	NR	Soup	[71]
Macaúbas, Bahía	2006	13	2 (15.3%)	[1.92–45.45]	NR	Cane sugar juice	[72]
Pará	2007	25	0 (0%)	[0.00–13.72]	NR	Açaí	[73]
Santarem, Manaus, Coarí	2006–2007	4	0 (0%)	[0.00–60.24]	NR	Açaí	[54]
Coarí, Amazonas	2007	25	0 (0%)	[0.00–13.72]	TcIII	NR	[74]
Belém, Pará	2007	4	0 (0%)	[0.00–60.24]	NR	Açaí	[75]
Belém, Pará	2010	1	0 (0%)	[0.00–97.50]	NR	Açaí	[76]
Río Negro, Aazonas	2010	17	0 (0%)	[0.00–19.51]	NR	Açaí	[77]
Carauarí, Amazonas (Unpublished)	2011	12	0 (0%)	[0.00–26.46]	NR	Açaí	[78]
Pará	2006–2012	668	20 (2.9%)	[1.84–4.59]	TcI(Zimodeme 1); TcIV (Zimodeme 3)	NR	[79]
Tocantis	2008–2014	22	1 (4.5%)	[0.12–22.84]	NR	Açaí, bacaba	Tocantis Health Secretary
Pará	2014	118	1 (0.84%)	[0.02–4.63]	NR	NR	Pará Health Secretary
Rio Grande Do Norte	2015	18	3 (16.6%)	[3.58–41.42]	NR	NR	[80]
Pará	2015	199	2 (1%)	[0.12–3.58]	NR	NR	Pará Health secretary
Pará	2016	273	6 (2.1%)	[0.81–4.72]	NR	NR	Pará Health secretary
Pará	2016	1	1 (100%)	[2.50–100.0]	NR		[81]
Acre	2009–2017	43	4 (9.3%)	[2.59–22.14]	NR	Açaí	Acre Health Secretary
Amazonas	2015–2017	24	0 (0%)	[0.00–14.25]	NR	Açaí	Amazonas Health Secretary
Pará	2017	211	1 (0.47%)	[0.01–2.61]	NR	NR	Pará Health Secretary
Marimarituba, Cachoeira do Aura, Pará	2016–2017	15	1 (6.66%)	[0.17–31.95] *	TcI;TcIV	Bacaba, Pataua	[82]
Several locations	2004–2022	147	0 (0%)	[0.00–2.48] *	TcIII/TcIV(zimodeme 3); TcIV	Açaí, Pataua	[83]
Ipixuna, Pará	2021	6	0(0%)	[0.00–45.93] *	NR	Açaí	[84]
**Colombia**							
Lebrija, Santander	2008	10	2 (20%)	[2.52–55.61]	TcI	Orange juice	[85]
Santander	2008–2009	20	3 (15%)	[3.21–37.89]	NR	NR	[86]
Antioquía	2010	11	1 (9.09%)	[0.23–41.28]	NR	NR	[87]
Aguachica, Cesar	2010	11	0 (0%)	[0.00–28.49]	TcI	NR	[88]
Casanare	2014	40	2 (0.5%)	[0.61–16.92]	NR	NR	[89]
Casanare, sucre, Antioquia, Atlantico, cesar, chocó	2019	124	10 (8.24%)	[3.27–12.86] *	TcI;TcII: TcIV	NR	[90]
César	2021	9	2 (22.2%)	[0.00–49.38] *	NR	NR	[91]
Cubará, Boyacá	2021	10	0 (0%)	[0.00–30.0] *	TcI; TcII	NR	[92]
Miraflores, Boyacá	2021	1	0 (0%)	[0.00–97.50] *	NR	NR	[93]
**French Guiana**							
Cayena	2005	8	0 (0%)	[0.00–36.94]	TcI	Palm juice	[51]
**Venezuela**							
Caracas	2007	1	0 (0%)	[0.00–97.50]	TcI	NR	[94]
Caracas	2007	103	1 (0.97%)	[0.02–5.29]	NR	Guava juice	[95]
Caracas (unpublished)	2008	3	0 (0%)	[0.00–70.76]	NR	NR	[18]
Chichiriviche, Caracaya	2009	88	4 (4.5%)	[1.25–11.23]	NR	NR	[96]
Chichiriviche, Caracaya	2009	89	5 (5.6%)	[1.85–12.63]	TcI	Guava juice	[47]
Táchira	2010	6	1 (16.6%)	[0.42–64.12]	NR	NR	[19]
Caracas	2012	4	0 (0%)	[0.00–60.24] *	NR	NR	[18]
Mérida	2013	5	1 (20%)	[0.51–71.64]	NR	NR	[97]
Mirimire, Falcón	2013	8	1 (12.5%)	[0.32–52.65]	NR	Mango juice	[18]
El Guapo, Miranda (unpublished)	2014	3	0 (0%)	[0.00–70.76]	NR	Pumarosa juice	[18]
Táchira (unpublished)	2014	5	0 (0%)	[0.00–52.18]	NR	NR	[18]

NR: Not Reported; *: calculated by us.

**Table 2:** Sources of infection and strategies for the prevention of oral Chagas disease outbreaks in South América.

Contaminated Food	Prevention
Açai Pulp	Heating 43 °C × 20 min/Pasteurization
Sugar Cane Juice	Pasteurization
Tropical Fruits Juice	Pasteurization
Palm Juice	Pasteurization
Wild Meat	Freezing/Complete cooking (internal temperature > 65 °C)

## Data Availability

The original contributions presented in this study are included in the article. Further inquiries can be directed to the corresponding author.
